# Association of the systemic immuno-inflammation index, neutrophil-to-lymphocyte ratio, and platelet-to-lymphocyte ratio with diabetic microvascular complications

**DOI:** 10.3389/fendo.2024.1367376

**Published:** 2024-04-10

**Authors:** Jiahang Li, Xueying Wang, Wenjing Jia, Kai Wang, Wenju Wang, Weibo Diao, Feiya Ou, Jing Ma, Yan Yang

**Affiliations:** ^1^ Department of Pharmacy, The Second Affiliated Hospital of Air Force Medical University, Xi’an, China; ^2^ Department of Traditional Chinese Medicine, The First Affiliated Hospital-of Air Force Medical University, Xi’an, China; ^3^ Department of Pharmacy, The Hospital of Traditional Chinese Medicine in Changwu Country, Changwu, China; ^4^ Department of Pharmacy, Sanya Rehabilitation and Recuperation Center, Joint Logistics Support Force, People's Liberation Army, Sanya, China

**Keywords:** T2DM, diabetic microvascular complications, SII, NLR, PLR

## Abstract

**Background:**

The systemic immuno-inflammation index (SII), neutrophil-to-lymphocyte ratio (NLR), and platelet-to-lymphocyte ratio (PLR) are widely used and have been shown to be predictive indicators of various diseases. Diabetic nephropathy (DN), retinopathy (DR), and peripheral neuropathy (DPN) are the most prominent and common microvascular complications, which have seriously negative impacts on patients, families, and society. Exploring the associations with these three indicators and diabetic microvascular complications are the main purpose.

**Methods:**

There were 1058 individuals with type 2 diabetes mellitus (T2DM) in this retrospective cross-sectional study. SII, NLR, and PLR were calculated. The diseases were diagnosed by endocrinologists. Logistic regression and subgroup analysis were applied to evaluate the association between SII, NLP, and PLR and diabetic microvascular complications.

**Results:**

SII, NLR, and PLR were significantly associated with the risk of DN [odds ratios (ORs): 1.52, 1.71, and 1.60, respectively] and DR [ORs: 1.57, 1.79, and 1.55, respectively] by multivariate logistic regression. When NLR ≥2.66, the OR was significantly higher for the risk of DPN (OR: 1.985, 95% confidence interval: 1.29–3.05). Subgroup analysis showed no significant positive associations across different demographics and comorbidities, including sex, age, hypertension, HbA1c (glycated hemoglobin), and dyslipidemia.

**Conclusion:**

This study found a positive relationship between NLR and DN, DR, and DPN. In contrast, SII and PLR were found to be only associated with DN and DR. Therefore, for the diagnosis of diabetic microvascular complications, SII, NLR and PLR are highly valuable.

## Introduction

Diabetes is a metabolic disease characterized by chronic hyperglycemia, and it currently lacks a complete and definitive cure due to a variety of factors. Currently, about 529 million people worldwide suffer from diabetes, and this number continues to rise annually. Type 2 diabetes mellitus (T2DM) is the main type of diabetes, accounting for about 90% of all diabetes patients (1). In China, about 140 million people have diabetes (2); however, the rate of diabetes treatment, prevention, and control in China is less than 50% (3, 4). The rising incidence of diabetes and decreasing control rates contribute to the growing prevalence of diabetic microvascular complications. Diabetic microvascular complications are a common and specific type of diabetes complication characterized by abnormal growth and leakage of microvessels, leading to local edema and the impairment of tissue function. Diabetic nephropathy (DN), diabetic peripheral neuropathy (DPN), and diabetic retinopathy (DR) are the most prominent and common microvascular complications. Approximately one-third of diabetic patients suffer from nephropathy or retinopathy, while two-thirds are diagnosed with peripheral neuropathy (5, 6). DN, DR, and DPN are major risk factors for end-stage renal disease, adult blindness, and non-traumatic amputation, respectively (7–9). These complications are also the primary causes of death and disability, seriously affecting the patients’ health, imposing a heavy burden on individuals and society, and emerging as a public health problem for the entire community. As no effective treatment exists to cure diabetic microvascular complications completely, early screening and detection remain particularly important.

The pathogenesis of diabetic microvascular complications is complex and still poorly defined. However, inflammatory cells and the cytokines have been shown to play an essential role. The systemic immuno-inflammation index (SII), neutrophil-to-lymphocyte ratio (NLR), and platelet-to-lymphocyte ratio (PLR) are currently utilized in the diagnosis of infectious diseases, cardiovascular diseases, tumors and other medical conditions because of their calculation simplicity and easy accessibility (10). SII is an indicator that characterizes systemic inflammation and the immune response, and considers neutrophils, lymphocytes, and platelets in its calculation. In 2014, Hu et al. first argued that SII could effectively predict the prognosis of patients with hepatocellular carcinoma and could be used as an important diagnostic indicator (11). Subsequently, numerous researches have linked SII to various diseases including tumors, infectious diseases, and cardiovascular diseases (12). NLR is a simple ratio of neutrophils to lymphocytes, and it is a potential biomarker reflecting the dysregulation of the immune response. The infectious diseases, autoimmune diseases, tumors, and surgical recovery have been found to be strongly associated with NLR (10, 13). NLR may also be considered a powerful prognostic marker for disease severity and mortality. Separately, PLR is a ratio of platelets to lymphocytes that represents the relationship between platelet and lymphocyte levels. In recent years, PLR has emerged as an inflammatory marker derived to assess many inflammatory conditions and cardiovascular diseases. More and more studies have shown that high PLRs can reflect the degrees of inflammation, platelet activation, and atherosclerosis (14, 15). Diabetic microvascular complications are highly associated with chronic inflammation (16, 17), but their associations with SII, NLR, PLR remain highly controversial (18, 19). Therefore, in order to investigate the association between SII, NLR, PLR and diabetic microvascular complications, we designed this cross-sectional study.

## Methods

### Data collection

**Figure 1:**
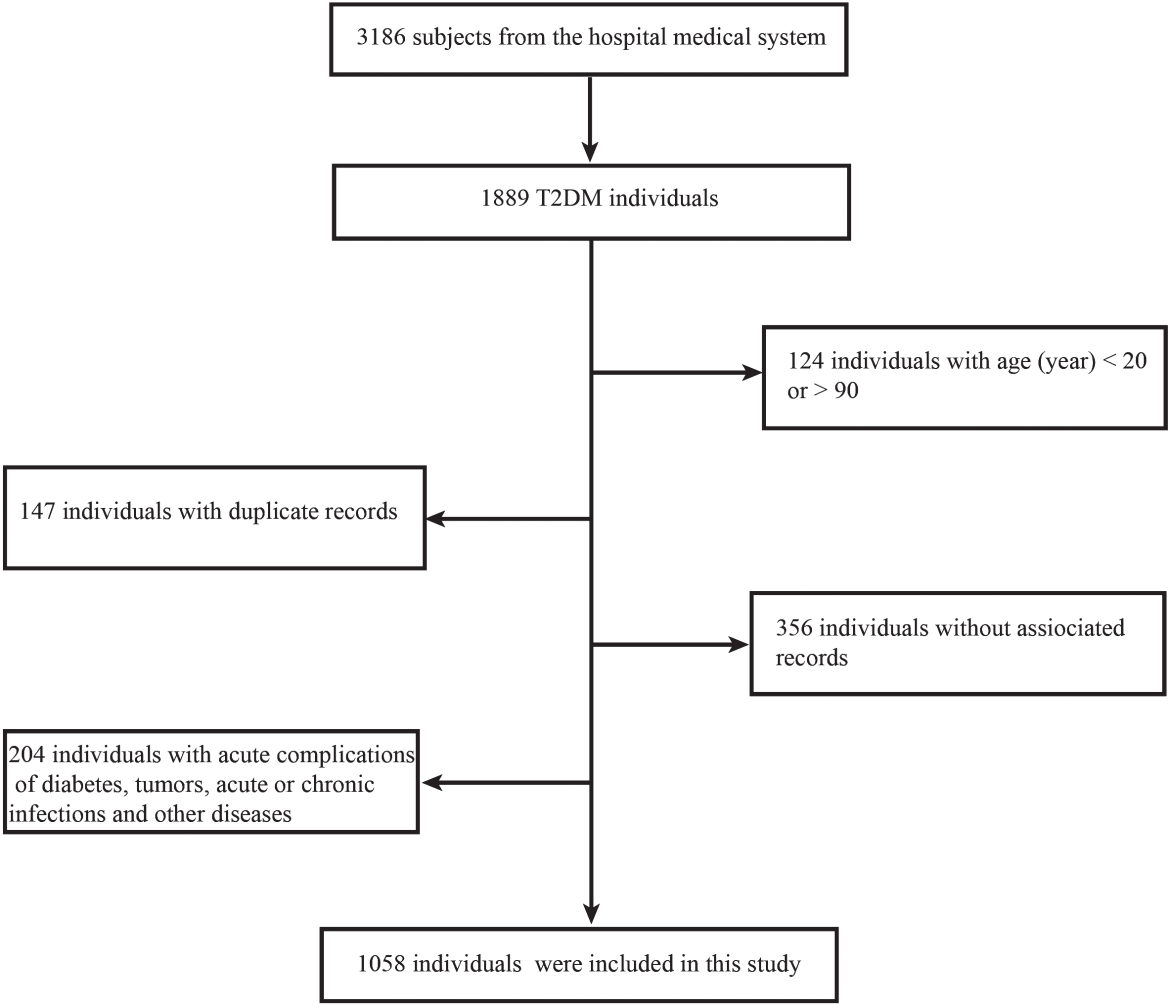
Flow chart for patient enrollment.

All of the data used in this study were sourced from the Endocrinology Department of Tangdu Hospital in China, with a time restriction of 2020–2023. Initially, a total of 3,186 data were collected during this cross-sectional retrospective study. After applying some exclusion criteria, a total of 1,058 effective data were made available. Inclusion criteria include: 1. a clear diagnosis of T2DM; 2. age range of 20–90 years; 3. the availability of relevant and complete medical data; and 4. an absence of acute complications of diabetes, tumors, acute or chronic infections, and other diseases that affect blood cell counts (**Figure 1**). The patients’ medical information included their basic personal information, disease history, medication history, and various laboratory tests performed during hospitalization. All blood cells in this study were counted using an automated hematology analysis device (XN9000; Sysmex, Kobe, Japan). NLR, SII, and PLR were calculated according to the neutrophil count/lymphocyte count, platelet count **×** neutrophil count/lymphocyte count, and platelet count/lymphocyte count, respectively.

### Definitions and groups

T2DM, hypertension, and hyperlipidemia were diagnosed by endocrinologists based on the latest guidelines (2, 12, 20), as described in detail in our previous articles (21, 22). The diagnosis of diabetic microvascular complications is firstly caused by diabetes, excluding the other primary causes. DN was defined as the presence of urinary albumin/creatinine ratio (ACR) of >30 mg/g and/or a progressive decrease in glomerular filtration rate (<60 mL/min/1.73 m2) (23–25). DR was diagnosed using grading criteria developed by the International Academy of Ophthalmology in 2002, which included diabetic macular edema in the management of DR (2). The diagnosis of DPN was primarily related to sensations in the limb, including pain, numbness, burning, and loss of protective sensation (26, 27).

According to the type of microvascular complications, all T2DM patients were categorized into the “no microvascular complications” (Non-MC) group or the microvascular complications group (DN group, DR group, or DPN group). In DN group, DR group, or DPN group, the patients may have one or more microvascular complications, and one patient may be in DN group, DR group, or DPN group at the same time. In addition, they were categorized into groups 0, 1, 2, or 3 depending on the number of microvascular complications. Mean values of NLR, SII, and PLR were used as cutoff values to analyze differences between each group.

### Statistical analysis

Qualitative and quantitative data are described in the form of numbers (percentages) and mean ± standard deviation values, respectively. Non-normally distributed quantitative data were expressed as the median and quantile spacing [M (P25%, P75%)]. Comparisons between two groups were conducted with the chi-squared test (for qualitative data), independent-samples t-test or Mann-Whitney U test (for quantitative data). A P-value <0.05 was considered to indicate significant difference in all statistical comparisons. All analysis was performed by SPSS Statistics 26 (IBM Corporation, Armonk, NY, USA).

The relationships between SII, NLR, and PLR and microvascular complications were evaluated by applying multiple logistic regression. They were also investigated by subgroup analysis according to sex, age, hypertension, dyslipidemia, and HbA1c (%) level. SII, NLR, and PLR values were divided into quartile groups according to the following values, quartile 1 (Q1): ≤267.41, ≤1.43, and ≤88.84; quartile 2 (Q2): 267.42–382.98, 1.44–1.89, and 88.85–112.28; quartile 3 (Q3): 382.99–552.86, 1.90–2.65, and 112.29–143.10; and quartile 4 (Q4): ≥552.87, ≥2.66, and ≥143.11, respectively. Multiple logistic regression was used to analyze the relationships between different SII, NLR, and PLR levels and microvascular complications.

## Results

### Baseline individual characteristics

There were 1058 enrollment individuals (337 women and 721 men). The mean HbA1c (%) level was 8.59 ± 2.20, and the duration of T2DM was 9.32 ± 7.10 years. The mean age was 54.67 ± 12.86 years. The numbers of patients with DN, DR, and DPN were 407 (38.47%), 260 (24.57%), and 340 (32.14%), respectively. In addition, 481 (45.46%) patients had hypertension and 842 (79.58%) had dyslipidemia. The mean values of NLR, PLR, and SII were 2.33 ± 2.25, 122.54 ± 53.55, and 489.14 ± 499.97, respectively. All data were presented in **Table 1**.

**Table 1:** Baseline individual characteristics.

Variables	Patients (1058)
Gender (Female/Male)	337/721
Age (years)	54.67 ± 12.86
≥55	598 (56.52%)
HbA1c (%)	8.59 ± 2.20
≥7	778 (73.53%)
Diabetic duration (years)	9.32 ± 7.10
≥10	475 (44.90%)
BMI (kg/m^2^)	26.04 ± 3.74
≥24	307 (29.02%)
DN	407 (38.47%)
DR	260 (24.57%)
DPN	340 (32.14%)
Hypertension	481 (45.46%)
Dyslipidemia	842 (79.58%)
Lymphocytes (10^9^/L)	2.01 ± 5.02
Neutrophils (10^9^/L)	3.89 ± 3.61
Platelets (10^9^/L)	208.62 ± 65.61
Monocytes (10^9^/L)	0.47 ± 0.33
BUN (mmol/L)	6.21 ± 9.07
Creatinine (umol/L)	68.25 ± 52.00
Uric acid (umol/L)	324.57 ± 90.10
eGFR (mL/min/1.73m^2^)	102.01 ± 23.84
NLR	2.33 ± 2.25
PLR	122.54 ± 53.55
SII	489.14 ± 499.97

BMI, body mass index; BUN, blood urea nitrogen; DN, diabetic nephropathy; DR, diabetic retinopathy; DPN, diabetic peripheral neuropathy; eGFR, estimated glomerular filtration rate; HbA1c, glycated hemoglobin; T2DM, type 2 diabetes mellitus; NLR, neutrophil-to-lymphocyte ratio; PLR, platelet-to-lymphocyte ratio; SII, systemic immuno-inflammation index.

### The comparisons between different groups

As shown in **Table 2**, the number of patients in the Non-MC, DN, DR, and DPN groups were 357 (33.74%), 407 (38.47%), 260 (24.57%), and 340 (32.14%), respectively. Age, duration of disease, the number of patients with hypertension or dyslipidemia, urea nitrogen, blood creatinine, eGFR, NLR, and SII showed significant increases in the microvascular complication groups. In particular, NLRs were 2.04 ± 1.23, 2.63 ± 2.80, 2.48 ± 1.61, and 2.45 ± 2.69 in the Non-MC, DN, DR, and DPN groups, respectively. In the Non-MC, DN, DR, and DPN groups, the SII values were 435.53 ± 277.41, 570.80 ± 672.64, 503.94 ± 370.84, and 487.04 ± 582.33, respectively. Furthermore, in the diabetic microvascular complication groups, the proportions of participants with high values of SII, NLR, and PLR were significantly greater.

**Table 2:** Characteristics in different groups.

Variables	Non-MC group N=357	DN group N=407	DR group N=260	DPN group N=340
Gender (Female/Male)	118/239	104/303	88/172	119/221
Age (years)	49.00 (37.00, 58.00)	58.00 (49.00, 65.00) ^**^	60.00 (55.00, 69.00) ^***^	59.00 (53.00, 67.00) ^***^
≥55	129 (36.13%)	250 (61.43%)^***^	191 (73.46%)^***^	259 (76.18%)^***^
HbA1c (%)	8.70 (7.00, 10.60)	8.20 (6.90, 10.10)	7.70 (6.80, 9.50)	7.90 (6.70, 9.80)
≥7	266 74.51%)	305 (74.94%)	191(73.46%)	240 (70.59%)
Diabetic duration (years)	4.00 (1.00, 8.00)	10.00 (5.00, 17.00) ^***^	12.00 (5.50, 19.50) ^***^	10.00 (6.00, 16.00) ^***^
≥10	73 (20.45%)	227 (55.77%)^***^	180 (69.23%)^***^	233(68.53%)^***^
BMI (kg/m^2^)	26.08 ± 4.07	26.45 ± 3.67	25.64 ± 3.39	25.79 ± 3.60
≥24	98 (27.45%)	107 (26.29%)	87 (33.46%)	117 (34.41%)
Hypertension	90 (25.21%)	258 (63.39%)^***^	167 (64.23%)^***^	174 (51.18%)^***^
Dyslipidemia	220 (61.62%)	361 (88.70%)^***^	232 (89.23%)^***^	306 (90.00%)^***^
Lymphocytes (10^9^/L)	2.45 ± 8.60	1.81 ± 0.60	1.73 ± 0.57	1.74 ± 0.57
Neutrophils (10^9^/L)	3.62 ± 1.42	4.27 ± 4.34^*^	3.98 ± 3.24^*^	3.87 ± 4.55
Platelets (10^9^/L)	215.47 ± 61.86	212.54 ± 70.01	202.07 ± 66.11	196.45 ± 64.79
Monocytes (10^9^/L)	0.47 ± 0.42	0.49 ± 0.30	0.46 ± 0.14	0.48 ± 0.38
Creatinine (umol/L)	57.11 ± 14.20	84.44 ± 74.90^***^	86.80 ± 86.39^***^	71.73 ± 74.25^***^
BUN (mmol/L)	5.31 ± 1.58	6.84 ± 3.30^***^	7.14 ± 3.47^***^	6.93 ± 15.64^*^
Uric acid (umol/L)	321.09 ± 86.67	342.90 ± 97.03^***^	334.22 ± 91.35	313.32 ± 82.42
eGFR (mL/min/1.73m^2^)	113.42 (102.16, 124.06)	99.28 (80.11, 112.09) ^***^	102.40 (91.74, 108.47) ^***^	104.43 (95.36, 113.42) ^*^
SII	435.53 ± 277.41	570.80 ± 672.64^***^	503.94 ± 370.84^*^	487.04 ± 582.33^*^
≥489.14	112 (31.37%)	169 (41.52%)^**^	104 (40.00%)^*^	105 (30.88%)
NLR	2.04 ± 1.23	2.63 ± 2.80^***^	2.48 ± 1.61^*^	2.45 ± 2.69^*^
≥2.33	88 (24.65%)	175 (43.00%)^***^	116 (44.62%)^***^	121 (35.59%)^**^
PLR	118.36 ± 49.94	127.51 ± 56.07	126.03 ± 52.61	122.20 ± 53.87
≥122.54	127 (35.57%)	186 (45.70%)^**^	121 (46.54%)^**^	137 (40.29%)

DN group including the patients of DN (211), DN+DR (88), DN+DPN (49), DN+DR+DPN (59).

DR group including the patients of DR (62), DN+DR (88), DR+DPN (51), DN+DR+DPN (59).

DPN group including the patients of DPN (181), DN+DPN (49), DPN+DR (51), DN+DR+DPN (59).

DN group, DR group, or DPN group vs. Non-MC group, ^*^P < 0.05, ^**^P < 0.01, ^***^P < 0.001.

The numbers of participants in groups 0, 1, 2, and 3 were 357 (33.74%), 454 (42.91%), 188 (17.77%), and 59 (5.58%), respectively. The values of SII, NLR, and PLR also increased (SII: 435.53 ± 277.41, 501.09 ± 559.08, 543.30 ± 669.97, and 548.90 ± 345.11; NLR: 2.04 ± 1.23, 2.42 ± 2.71, 2.55 ± 2.65, and 2.79 ± 1.42; PLR: 118.36 ± 49.94, 123.63 ± 55.81, 126.06 ± 56.37, and 128.15 ± 46.74, respectively) with number of complications. In particular, there was a significant difference for NLR and SII. The proportions of participants with high values of SII, NLR, and PLR also increased significantly (**Table 3**).

**Table 3:** Differences between various complication groups.

Variables	Number of complications			
	0	1	2	3
N (%)	357 (33.74%)	454 (42.91%)	188 (17.77%)	59 (5.58%)
SII	435.53 ± 277.41	501.09 ± 559.08^*^	543.30 ± 669.97^**^	548.90 ± 345.11^*^
≥489.14	112 (31.37%)	141 (31.06%)	78 (41.49%)^*^	27 (45.76%)^*^
NLR	2.04 ± 1.23	2.42 ± 2.71^*^	2.55 ± 2.65^*^	2.79 ± 1.42^*^
≥2.33	88 (24.65%)	150 (33.04%)^***^	80 (42.55%)^***^	34 (57.63%)^***^
PLR	118.36 ± 49.94	123.63 ± 55.81	126.06 ± 56.37	128.15 ± 46.74^*^
≥122.54	127 (35.57%)	179 (39.43%)^*^	86 (45.74%)^*^	31 (52.54%)^*^

Group 1, Group 2, or Group 3 vs. Group 0, ^*^P < 0.05, ^**^P < 0.01, ^***^P < 0.001.

### Association between SII, NLR, and PLR and microvascular complications

To analyze the relationship between SII, NLR, and PLR and diabetic microvascular complications, multivariate logistic regression was applied. SII, NLR, and PLR were significantly associated with the risk of DN (odds ratio [OR]: 1.52, 1.71, 1.60, respectively) and DR (OR: 1.57, 1.79, 1.55, respectively) after adjustment for age, body mass index, sex, diabetic duration, hypertension, dyslipidemia, and HbA1c (**Table 4**).

**Table 4:** Association between SII, NLR, and PLR and microvascular complications.

Variables	DN		DR		DPN	
	OR (95% CI)	*P*-value	OR (95% CI)	*P-*value	OR (95% CI)	*P-*value
SII ≥489.14	1.52 (1.07–2.16)	0.021	1.57 (1.06–2.34)	0.025	0.81 (0.54–1.21)	0.299
NLR ≥2.33	1.71 (1.19–2.48)	0.004	1.79 (1.17–2.74)	0.008	1.05 (0.67–1.57)	0.827
PLR ≥122.54	1.60 (1.13–2.28)	0.008	1.55 (1.03–2.34)	0.038	1.02 (0.69–1.49)	0.938

OR, odds ratio; CI, confidence interval.

**Figure 2:**
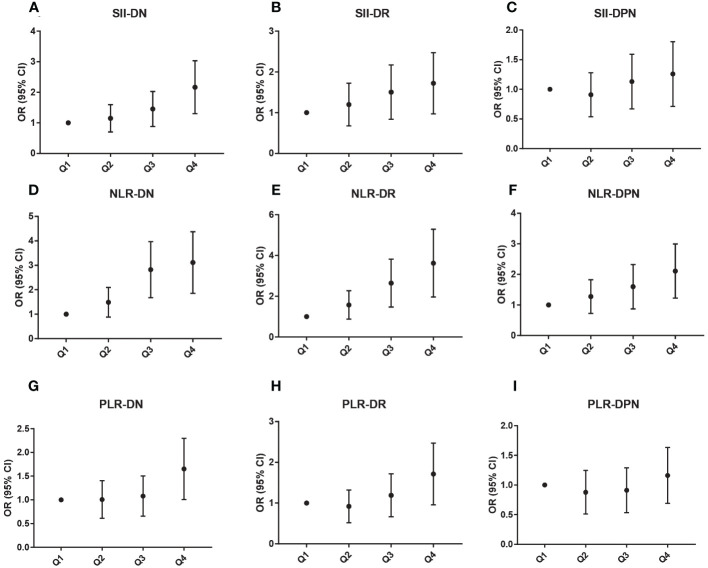
Association between different SII **(A–C)**, NLR **(D–F)**, and PLR **(G–I)** levels and microvascular complications.

SII, NLR, and PLR values were divided into quartile groups to further analyze the association with different levels of SII, NLR, and PLR and diabetic microvascular complications. **Figure 2** showed that the ORs of DN, DR, and DPN gradually increased with rising levels of SII, NLR, and PLR. In the NLR Q3 and Q4 groups, the ORs were significantly higher for DN (OR: 2.67, 95% CI: 1.76–4.04; OR: 2.94, 95% CI: 1.95–4.45) and DR (OR: 2.47, 95% CI: 1.57–3.89; OR: 3.37, 95% CI: 2.10–5.40). The ORs were significantly higher for DN (OR: 2.05, 95% CI: 1.37–3.08) and DR (OR: 1.61, 95% CI: 1.03–2.52) in the SII Q4 group. Moreover, the OR was significantly higher for DPN (OR: 1.99, 95% CI: 1.29–3.05) in the NLR Q4 group. Finally, in the PLR Q4 group, the ORs were significantly higher for DN (OR: 1.57, 95% CI: 1.05–2.34) and DR (OR: 1.60, 95% CI: 1.02–2.52).

To analyze the relationship of each combination of diabetic complications with NLR, PLR, and SII, we grouped each patient according to the type of complication, and each patient could only enter one group (**Table 5**). The regression analysis revealed that the ORs of NLR were significantly higher for DN, DR, DPN, DN+DR, DN+DPN, and DN+DR+DPN (ORs: 1.76, 1.93, 1.77, 2.97, 2.49, and 4.16). The ORs of PLR were significantly higher for DN, DR, DN+DR, and DN+DR+DPN (ORs: 1.45, 1.81, 1.73, and 2.01). Moreover, the ORs of SII were significantly higher for DN, DR, DN+DR, and DN+DR+DPN (ORs: 1.51, 1.47, 2.00, and 1.85).

**Table 5:** Association between SII, NLR, and PLR and each combination of microvascular complications.

	Event, n	NLR		PLR		SII	
		OR (95% CI)	*P-*value	OR (95% CI)	*P*-value	OR (95% CI)	*P*-value
DN	211	1.76 (1.21-2.54)	0.003	1.45 (1.15-1.91)	0.034	1.51 (1.00-2.28)	0.048
DR	62	1.93 (1.10-3.40)	0.022	1.81 (1.06-2.77)	0.038	1.47 (1.14-2.03)	0.014
DPN	181	1.74 (1.16-2.70)	0.016	0.97 (0.67-1.41)	0.860	1.34 (0.94-1.91)	0.112
DN+DR	88	2.97 (1.72-4.52)	<0.001	1.73 (1.08-2.77)	0.022	2.00 (1.24-3.21)	0.004
DN+DPN	49	2.49 (1.35-4.59)	0.003	1.48 (0.81-2.70)	0.206	1.51 (0.82-2.78)	0.188
DR+DPN	51	1.40 (0.74-2.65)	0.304	1.27 (0.70-2.31)	0.407	1.31 (0.83-1.88)	0.261
DN+DR+DPN	59	4.16 (2.35-7.35)	<0.001	2.01 (1.15-3.49)	0.014	1.85 (1.06-3.23)	0.032

### Subgroup analysis

**Figure 3:**
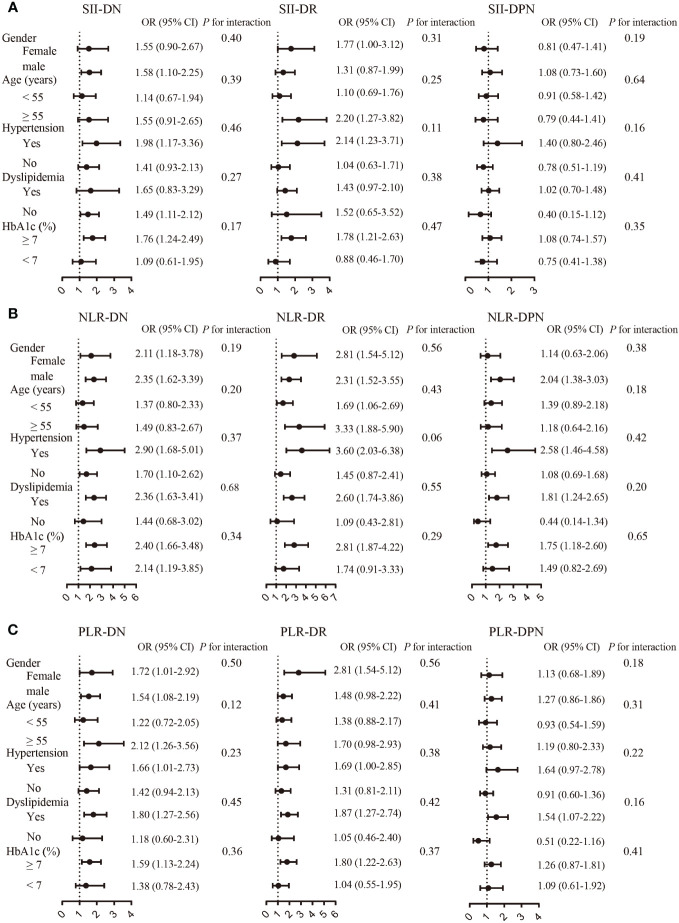
Association between SII **(A)**, NLR **(B)**, and PLR **(C)** and microvascular complications by subgroup analysis.

The subgroup analysis demonstrated differences between various subgroups in the associations of SII, NLR, and PLR with diabetic microvascular complications (**Figure 3**). Overall, the relationship between SII, NLR, and PLR and diabetic microvascular complications was stronger among patients with hypertension, dyslipidemia, or hyperglycemia. However, there was no significant differences between the sex, age, hypertension, HbA1C, and dyslipidemia subgroups in this relationship as determined through the interaction test.

## Discussion

In this study, the SII, NLR and PLR were collected together with DN, DR and DPN to analyze the relationship for the first time. The cross-sectional study included 1058 T2DM patients. Ultimately, the results revealed the positive relationship between SII, NLR, and PLR and diabetic microvascular complications.

The prevalence of diabetes has continued to rise with the aging of the population, changing lifestyles, progression of the urbanization process, and the increased prevalence of various metabolic diseases. Previously, the Global Burden of Disease Study updated the latest research findings on the global burden of diabetes for 1990–2021 and made predictions for 2050. Expectedly, there will be 1.31 billion diabetic patients in 2050 (1). Poor rates of diagnosis, treatment, and control of diabetes have resulted in a gradual rise in the number of patients with diabetic microvascular complications (21). DN, DR, and DPN emerge as the most prominent and common diabetic microvascular complications, affecting approximately more than 25% of diabetic patients (5, 28). Globally, the diabetic microvascular complication prevalence varies significantly. It was approximately 20% in patients with a diabetic duration of <1 year and nearly 50% in patients with a diabetic duration of >10 years from the 3B Study in China (2). The prevalence of microvascular complications in T2DM patients was 18.8% in the world, which was greatest in Europe (23.5%) and lowest in Africa (14.5%), according to the DISCOVER study conducted from 2014–2019 (6, 29).This study showed that the prevalence were 38.47%, 24.57%, and 32.14% for DN, DR, and DPN, respectively. The results are higher than those documented in Europe and Africa. The reason for this might be the ethnicity and the lower diagnosis, treatment, and control rates of T2DM in China. DN, DR, and DPN are important causes of death and disability in diabetic patients. Therefore, early diagnosis and screening are especially important for proper management of diabetic microvascular complications.

It is well known that the hyperglycemia and diabetic duration serve as major risk factors for diabetic microvascular complications. However, the long-term chronic inflammatory response also accelerates the progression of diabetic microvascular complications (9, 30, 31). The diabetic microenvironment activates local and systemic inflammatory responses, contributing to the activation of a large number of inflammatory cells (16, 17). The chronic inflammatory microenvironment in turn leads to microvascular endothelial cell damage and apoptosis, further exacerbating diabetic microvascular complications (32). Neutrophils, lymphocytes, and platelets are parts of the immune system that regulate natural and adaptive immunity and major players in the pathogenesis of diabetic microvascular complications. Recent studies found an increase in neutrophil counts in patients with DN and DR, and the neutrophil counts were also correlated with the development of DN and DR (19, 33). In this study, we found neutrophil counts to have significantly increased in both the DN and DR groups, which aligns with the results of the former research. The finding suggested that high levels of neutrophils are the risk factor for DN and DR.

SII, NLR, and PLR, which consider neutrophils, lymphocytes, and platelets, are currently used in the diagnosis due to their simplicity of calculation and ease of access (10). Since SII was found to be highly associated with the prognostic risk of hepatocellular carcinoma, as argued by Hu et al. in 2014, it has been shown that SII can serve as a risk factor for tumors, cardiovascular diseases, infectious diseases, metabolic diseases and others (11, 12). NLR is a reflection of the dynamic between neutrophils and lymphocytes in disease states, and now it has been widely used in a variety of medical fields. NLR serves as a marker of immune response to a variety of infectious and non-infectious triggers, and it can be used as an early warning sign for diseases such as tumors, atherosclerosis, infections, inflammation, and psychiatric disorders (34). PLR is the ratio of platelet counts to lymphocyte counts, and it is an inflammatory marker derived to assess many inflammatory diseases, tumors, and cardiovascular diseases in recent years (14, 15). However, the relationship between SII, NLR, and PLR and diabetic microvascular complications is controversial. A study with 1192 T2DM patients found that NLR was not associated with DN (19), whereas another study that recruited 4813 T2DM subjects concluded that NLR was associated with DN but not with DR (18). Other authors have concluded that NLR and PLR both relate to DR and DPN (35–37). Our results showed NLR was found to be associated with the risk of DN, DR, and DPN, while only high levels of SII and PLR were associated with the risk of DN and DR. Although the populations in our study and the previous studies were Chinese, the average level of age, eGFR, and lymphocyte were different. Multiple confounding variables and different conditions may have contributed to the differences in results. In addition, Duan et al. and Guo et al. thought high levels of SII and PLR were the risk factors for DN (38, 39). These results were consistent with ours. The positive association between NLR and diabetic microvascular complications is more significant in most of the studies, as evidenced by our results. The reason for this may be that the neutrophilic effect in chronic inflammation plays a more important role in the development of diabetic microvascular complications. Microvascular complications due to high glucose environment is associated with systemic and local inflammation, among which involve inflammatory cells. As indicators of the inflammatory response, neutrophils, lymphocytes, and platelets all play appropriate roles. In most studies, neutrophil counts are significantly elevated in patients with diabetic microvascular complications, whereas platelet and lymphocyte counts are not always significantly altered (19, 33, 40). The predominance of neutrophil counts and their sensitivity could explain this feature. This phenomenon may lead to the instability of the relationship between PLR, SII and diabetic microvascular complications.

Diabetic microvascular complications are mainly related to the duration of the diabetes and the glucose control. With the increasing of age and diabetic during, T2DM patients may have several complications at the same time. Therefore, it is necessary to analyze the relationship of each complication’ combination with NLR, PLR, and SII. In the complication’ combination, NLR was associated with the risk of DN, DR, DPN, DN+DR, DN+DPN, and DN+DR+DPN, while SII and PLR were associated with the risk of DN, DR, DN+DR, and DN+DR+DPN. From another perspective, this result revealed that DN and DR were more closely related to SII and PLR than DPN.

According to the best of our knowledge, the SII, NLR and PLR were taken together with DN, DR and DPN to analyze the relationship for the first time, in this study. The data from this study further revealed the relationship between SII, NLR, and PLR and the risk of DN, DR, and DPN, respectively. NLR, SII, and PLR might be considered in efforts to diagnose diabetic microvascular complications.

### The limitations

Notably, our results confirmed a positive association between NLR, SII, and PLR and diabetic microvascular complications. However, the study limitations are also clear. This is a single-center study, and factors such as individual enrollment, physician competence, and examination errors may have some data bias. In addition, only the association was evaluated in this cross-sectional study, as causation data are not available. This is a deficiency from the design. Therefore, the quantity of data was not large enough, and more data are still needed.

## Conclusion

This study found a positive relationship between NLR and DN, DR, and DPN, while only SII and PLR were associated with DN and DR. Therefore, for the diagnosis of diabetic microvascular complications, SII, NLR and PLR are highly valuable.

## Data availability statement

The original contributions presented in the study are included in the article/supplementary material. Further inquiries can be directed to the corresponding authors.

## Ethics statement

The studies involving humans were approved by The Ethics Committee of Tangdu Hospital (reference number: K202205-07). The studies were conducted in accordance with the local legislation and institutional requirements. The participants provided their written informed consent to participate in this study. Written informed consent was obtained from the individual(s) for the publication of any potentially identifiable images or data included in this article.

## Author contributions

JL: Investigation, Writing – original draft, Writing – review & editing, Data curation, Formal analysis, Software. XW: Writing – original draft, Writing – review & editing, Software. WJ: Data curation, Investigation, Writing – review & editing. KW: Data curation, Investigation, Writing – review & editing. WW: Data curation, Investigation, Writing – review & editing. WD: Data curation, Investigation, Writing – review & editing. FO: Data curation, Investigation, Writing – review & editing. JM: Resources, Validation, Writing – review & editing. YY: Writing – review & editing, Resources, Funding acquisition, Project administration.
